# Feasibility of repairing full-thickness skin defects by iPSC-derived epithelial stem cells seeded on a human acellular amniotic membrane

**DOI:** 10.1186/s13287-019-1234-9

**Published:** 2019-05-31

**Authors:** Huateng Zhou, Lixiang Wang, Cui Zhang, Jintao Hu, Jianlin Chen, Weibin Du, Fei Liu, Weifan Ren, Jinfu Wang, Renfu Quan

**Affiliations:** 1 Clinical Medical College, Zhejiang Chinese Medical University, Zhejiang, 310053 Hangzhou China; 2 Department of Orthopedic Surgery, Xiaoshan Traditional Chinese Medical Hospital, Zhejiang, 311200 Hangzhou China; 3 Laboratory of Stem Cells, Institute of Cell Biology, College of Life Sciences, Zhejiang University, Zhejiang, 310058 Hangzhou China; 4 Department of Chinese Medicine Rehabilitation, Xiushan People’s Hospital, Xiushan, Chongqing, 409900 China

**Keywords:** Skin defect, Induced pluripotent stem cells, CD200^+^/ITGA6^+^ epithelial stem cells, Hair follicle

## Abstract

**Background:**

Induced pluripotent stem cells (iPSCs) can generate epithelial stem cells (EpSCs) as seed cells for skin substitutes to repair skin defects. Here, we investigated the effects of a human acellular amniotic membrane (hAAM) combined with iPSC-derived CD200^+^/ITGA6^+^ EpSCs as a skin substitute on repairing skin defects in nude mice.

**Methods:**

Human urinary cells isolated from a healthy donor were reprogrammed into iPSCs and then induced into CD200^+^/ITGA6^+^ epithelial stem cells. Immunocytochemistry and RT-PCR were used to examine the characteristics of the induced epithelial stem cells. iPSC-derived EpSCs were cultured on a hAAM, and cytocompatibility of the composite was analyzed by CCK8 assays and scanning electron microscopy. Then, hAAMs combined with iPSC-derived EpSCs were transplanted onto skin defects of mice. The effects of this composite on skin repair were evaluated by immunohistochemistry.

**Results:**

The results showed that CD200^+^/ITGA6^+^ epithelial stem cells induced from iPSCs displayed the phenotypes of hair follicle stem cells. After seeding on the hAAM, iPSC-derived epithelial stem cells had the ability to proliferate. After transplantation, CD200^+^/ITGA6^+^ epithelial stem cells on the hAAM promoted the construction of hair follicles and interfollicular epidermis.

**Conclusions:**

These results indicated that transplantation of a hAAM combined with iPS-derived EpSCs is feasible to reconstruct skin and skin appendages, and may be a substantial reference for iPSC-based therapy for skin defects.

## Background

Skin is the largest organ of the human body and the barrier between internal and external environments. Because skin is located at the surface of the human body, it is vulnerable to injury. Each year, more than 70 million people suffer from skin defects and require surgical treatment [1], resulting in significant medical costs [2]. Moreover, large skin defects as a result of extensive burns or trauma may lead to infection, fluid loss, organ failure, and even death. A skin graft is considered to be the most effective therapy for the treatment of skin defects, which is limited because of the insufficient supply of autologous skin and the risks of xenografts [3]. Therefore, a skin substitute is believed to have great potential to repair skin defects. In particular, a full-thickness skin substitute consisting of scaffolds and cells can partly substitute the function of skin and accelerate skin healing. Cells play a vital role in skin substitutes. Many kinds of cells have been used as seed cells for skin substitutes, such as keratinocytes [4], mesenchymal stem cells [5], dermal fibroblasts [6], and others [7, 8]. However, cells with more potential to construct a skin substitute remain to be discovered.

Hair follicle stem cells play a crucial role in the process of skin reconstruction. They are not only involved in the construction of the interfollicular epithelium, but also in the formation of hair follicles and other skin appendages [9, 10]. These cells express cytokeratin 15 (Krt15) [11, 12], CD200, and integrin A6 (ITGA6) [13], are located in the niche of the hair follicle (bulge), and maintain a quiescent state (slow-cycling cells) before activation [14]. Some skin substitutes based on hair follicle stem cells have been studied [15–17]. However, it is still challenging to obtain enough pure hair follicle stem cells to construct skin substitutes. Takahashi et al. [18–20] first established induced pluripotent stem cells (iPSCs) generated from somatic cells by transduction of four reprogramming factors, which have properties resembling those of embryonic stem cells: the potential for unlimited proliferation and the capability of differentiation into cells of all three embryonic germ layers, such as pulmonary epithelial cells [21], cardiomyocytes [22], and keratinocytes [23]. Using methods to induce embryonic stem cells to differentiate into epidermal stem cells [24, 25], Bilousova et al. [23] induced iPSCs to differentiate into keratinocytes by regulating bone morphogenetic protein (BMP-4) and retinoic acid (RA) signaling pathways. Consequently, Yang et al. [26] induced iPS cells into epidermal stem cells expressing hair follicle-specific markers CD200 and ITGA6 by regulating the epidermal growth factor (EGF) signaling pathway. These cells not only expressed genes specific for hair follicle stem cells, but also had similar abilities to hair follicle stem cells: forming a new interfollicular epithelium and skin appendages such as hair follicles and sebaceous sweat glands. Therefore, these cells are termed as folliculogenic epithelial stem cells [26]. Because of the unlimited proliferation of iPS cells and great potential of hair follicle stem cells as seed cells for skin substitutes, iPSC-derived CD200^+^/ITGA6^+^ EpSCs may be promising seed cells for skin substitutes.

Here, we induced urine cell-derived iPSCs to differentiate into CD200^+^/ITGA6^+^ EpSCs and cultured these cells on a human acellular amniotic membrane (a promising skin substitute scaffold prepared by removal of epithelial cells from an amniotic membrane [27]) to form skin substitutes that were then transplanted onto skin defects of nude mice. The effects of iPSC-derived EpSCs on wound healing were then analyzed. We also assessed the feasibility of using iPSC-derived EpSCs as seed cells to construct skin substitutes to repair skin defects.

## Materials and methods

### Generation and analysis of iPSCs

The study was approved by the Ethics Committee of Zhejiang Health Bureau, Hangzhou, Zhejiang. Urine samples were collected from a 23-year-old healthy male donor after obtaining informed consent. Cells were isolated from the urine using a previously described method [28]. Briefly, 200 mL urine was collected from the donor and centrifuged at 400*g* for 10 min at room temperature. After discarding the supernatant, the residual urine sample (1 mL) was washed with 10 mL phosphate-buffered saline (PBS) containing 2.5 μg/mL amphotericin B (Solarbio, Beijing, China), 100 U/mL penicillin, and 100 μg/mL streptomycin (Solarbio). Cells were centrifuged again and then resuspended in 1 mL primary medium [1:1 mixture of high glucose Dulbecco’s modified Eagle’s medium (DMEM) (Life Technologies, Shanghai, China) with 10% (*v*/*v*) fetal bovine serum (FBS) (Gibco, Shanghai, China) and SingleQuot Kit CC-4127 renal epithelial cell growth medium (REGM; Lonza, Shanghai, China) supplemented with 2.5 μg/mL amphotericin B, 100 U/mL penicillin, and 100 μg/mL streptomycin (Gibco)]. Then, the cells were seeded in 6-well plates (Corning, Shanghai, China) coated with 0.1% gelatin (Sigma, Shanghai, China) and cultured at 37 °C in a humidified atmosphere with 5% CO_2_. Subsequently, 500 μL primary medium was added to the cells for the first 2 days, and half of the culture medium was renewed daily with RE proliferation medium [renal epithelial basal medium (REBM; Lonza) supplemented with SingleQuot Kit CC-4127 REGM]. When the first colonies were visible, the medium was replaced with RE proliferation medium. Upon reaching 90% confluence, the cells were passaged using TrypLE Express (Gibco) at a split ratio of 1:2 or 1:3. Cells from passage 3 were used to induce iPSCs.

To generate iPSCs, urinary cells were electrotransfected (Lonza) with an Amaxa Basic Nucleofector Kit for primary mammalian epithelial cells according to Program T-020 (Lonza). In each nucleofection, 1 × 10^6^ urinary cells were treated with 1 μg PCXLE-hOCT3/4-shp53-F (Plasmid #27077), 1 μg PCXLE-hSK (Plasmin #27078), 1 μg pCXLE-hUL (Plasmid #27080), and 1 μg pCXWB-EBNA1 (Plasmin #37624). All plasmids were obtained from Addgene (https://www.addgene.org/). Then, cells were seeded in a 6-well plate coated with 0.1% gelatin and cultured in urinary cell medium for 5–7 days. They were then transferred onto mouse embryonic fibroblasts and cultured in hES medium [DMEM/F12 (1:1) supplemented with 20% (*v*/*v*) knockout serum replacement (Gibco), 1% GlutaMAX, 1% nonessential amino acid (Gibco), 100 μM β-mercaptoethanol (Gibco), and bFGF (10 ng/mL, Thermo Fisher, Shanghai, China)] for 10–16 days while renewing the medium every day until iPSC colonies appeared. Individual colonies were collected using a glass needle and expanded in mTeSR1 medium (StemCell, Shanghai, China) in plates coated with Matrigel (Corning, Shanghai, China). The iPSCs were passaged every 3–5 days using TrypLE Express at a split ratio of 1:3–1:5.

For immunocytochemistry, cells were fixed for 15 min in 4% paraformaldehyde at room temperature, permeabilized with 0.25% (*v*/*v*) Triton X-100/PBS for 10 min, and then blocked with PBS containing 4% (*w*/*v*) bovine serum albumin (Amresco, Shanghai, China) for 1 h at room temperature. Incubations with primary antibodies were carried out overnight at 4 °C, and those with secondary antibodies were carried out for 30 min at room temperature. The primary antibodies were anti-OCT4 (1:350, rabbit polyclonal), anti-NANOG (1:1000, rabbit monoclonal), anti-SSEA (1:70, mouse monoclonal), anti-TRA-1-81 (1:100, mouse monoclonal), and anti-TRA-1-60 (1:100, mouse monoclonal). Secondary antibodies were goat anti-rabbit Alexa Fluor 488 or 568 and goat anti-mouse Alexa Fluor 647 (Abcam, Shanghai, China).

RNA extraction was performed according to the TRIzol™ Reagent protocol after collecting the cells, and RNA concentrations were measured by a Nanodrop (Thermo Scientific, Shanghai, China). cDNA was synthesized by a RevertAid First Strand cDNA Synthesis Kit (Thermo Scientific), according to the manufacturer’s instructions. PCR was performed as follows: 95 °C for 5 min, followed by 30 cycles at 94 °C for 30 s, primer annealing at 65 °C for 30 s, and 72 °C for 1 min. Primers were designed using Primer Premier 6.0 Demo and Oligo 7.36 Demo software as shown in Table 1. PCR products were electrophoresed on a 1.5% agarose gel and stained with ethidium bromide. Stained PCR products were visualized under ultraviolet light and photographed.

**Table 1:** Primer sequences for reverse transcription-polymerase chain reaction

Gene name	Forward primer (5′–3′)	Reverse primer (5′–3′)
OCT4	GACAGGGGGAGGGGAGGAGCTAGG	CCTCCCTCCAACCAGTTGCCCCAAAC
SOX2	GGGAAATGGAGGGGTGCAAAAGAGG	TTGCGTGAGTGTGGATGGGATTGGTG
KLF4	GAGGGAAGACCAGAATTCCCTTGA	AGAAC AA ACTCACCAAGCACCA
c-MYC	TGCACTGGAACTTACAA ACCCGA	TAA GCA GCT GCA AGG AGA GCCTTT
LGR5	GAGTTACGTCTTGCGGGAAAC	TGGGTACGTGTCTTAGCTGATTA
LGR6	AGCCCTGTGAGTACCTCTTTG	CAGCACCAGTCCATTGCAGA
TCF4	CAAGCACTGCCGACTACAATA	CCAGGCTGATTCATCCCACTG
CD200	ACAGCCCATAGTATCCCTTCAC	GATGCTGGTAACAGACGTGGT
ITGA6	ATGCACGCGGATCGAGTTTG	TTCCTGCTTCGTATTAACATGC
FZD2	GTGCCATCCTATCTCAGCTACA	CTGCATGTCTACCAAGTACGTG
DKK3	ACGAGTGCATCATCGACGAG	GCAGTCCCTCTGGTTGTCAC
CTNNB1	AAAGCGGCTGTTAGTCACTGG	CGAGTCATTGCATACTGTCCAT
LEF1	TGCCAAATATGAATAACGACCCA	GAGAAAAGTGCTCGTCACTGT
LHX2	ATGCTGTTCCACAGTCTGTCG	GCATGGTCGTCTCGGTGTC
KRT14	TGAGCCGCATTCTGAACGAG	GCAGTAGCGACCTTTGGTCT
KRT15	GACGGAGATCACAGACCTGAG	CTCCAGCCGTGTCTTTATGTC
KRT19	ACCAAGTTTGAGACGGAACAG	CCCTCAGCGTACTGATTTCCT
NANOG	AAGGTCCCGGTCAAGAAACAG	ATCCCTGCGTCACACCATTGC
REXO1	CCCTCCGTCCACATTTCCG	GCGATTCGCTTAGGGATGATG

To prepare cells expressing green fluorescent protein (GFP), a lentivirus was produced by transfection of 1.875 μg psPAX2, 2.5 μg pGIP2-EFG, and 0.625 μg PMD2.G into HEK293T cells. Then, iPSCs were infected with the lentivirus at a density of 1 × 10^5^ cells/well in a 6-well plate.

An alkaline phosphatase detection kit (Sigma) was used to detect alkaline phosphatase activity. For teratoma formation, 1 × 10^6^ iPSCs were injected subcutaneously into the flank of NSG mice (Slack, Shanghai, China). The presence of the three germ layer organizations in tumors was examined by histology.

### Generation and analysis of CD200^+^/ITGA6^+^ EpSCs derived from iPSCs

The protocol for induction of iPS cells into EpSCs was adapted from Yang et al. [26] with minor modifications. Briefly, iPSC colonies were grown for 2–3 days on Matrigel in mTeSR1 (StemCell) until 60% confluence. Then, cells were separated into small clusters by incubation with dispase II (0.2% in DMEM, Sigma-Aldrich, Shanghai, China) for 10–15 min (10–20 cells), which were washed twice with DMEM and cultured in a 6-well low-cluster plate (Corning Costar, NY, USA) in 2 mL hESC medium containing Y-27632 (MedChemExpress, Beijing, China) and 1 ng/mL bone morphogenetic protein 4 (BMP-4, Thermo Fisher) without bFGF for 1 day to form embryoid bodies (EBs). Then, the newly formed EBs were seeded onto mitomycin-C-treated (MedChemExpress) 3T3 fibroblasts and cultured in differentiation medium A [3:1 mixture of DMEM and Ham’s F-12 Nutrient Mixture (F12, Gibco) supplemented with 2% (*v*/*v*) fetal bovine serum (FBS, Gibco), 5 mg/mL insulin, 0.5 mg/mL hydrocortisone, 10^−10^ mol/L cholera toxin, 1.37 ng/mL triiodothyronine, 0.3 mmol/L l-ascorbic acid, 24 mg/mL adenine, and 1 mmol/L all-trans retinoic acid (RA)]. All supplements (unless specified otherwise) were purchased from Sigma-Aldrich. Then, the cells were cultured in differentiation medium B [differentiation medium A supplemented with 25 ng/mL BMP-4 and 20 ng/mL EGF (Thermo Fisher)] for 4 days. Finally, the cells were cultured in differentiation medium C [Defined Keratinocyte-SFM (D-KSFM, Invitrogen, Shanghai, China) with 5 mg/mL insulin, 0.2 mg/mL hydrocortisone, 10^−10^ mol/L cholera toxin, 1.37 ng/mL triiodothyronine, 0.3 mmol/L l-ascorbic acid, 10 mg/mL adenine, 1 mmol/L RA, 25 ng/mL BMP-4, and 20 ng mL^−1^ EGF (Thermo Fisher)] for 4 days. Subsequently, the differentiated cells were detached with dispase II. Some detached cells were further digested into single cells with TrypLE Express (ThermoFisher) and cultured on mitomycin-C-treated 3T3 fibroblasts again in differentiation medium D (Defined Keratinocyte-SFM with 1 ng/mL BMP-4 and 20 ng/mL EGF) for 7 days.

Immunocytochemistry was performed with primary antibodies against CD200 (1:200, mouse, monoclonal, ab23552), Cytokeratin 15 (Krt15) (1:200, mouse monoclonal, ab80522), Cytokeratin 19 (Krt19) (1:200, mouse monoclonal, ab52625), NANOG (1:200, rabbit monoclonal, ab109250), Integrin B1 (ITGB1) (1:200, rabbit monoclonal, ab134179), and Integrin A6 (ITGA6) (1:200, mouse monoclonal, ab20142) (Abcam) to characterize the cells differentiated from EBs. The secondary antibodies were goat anti-rabbit Alexa Fluor 568 and goat anti-mouse Alexa Fluor 647 (Abcam). Identifying and quantifying cell phenotypes was analyzed in image J 1.52 K (National Institutes of Health, Bethesda, MD, USA).

Flow cytometric analysis was performed to detect the number of Krt14^+^ and CD200^+^/ITGA6^+^ cells. Differentiated cells were detached and separated with TrypLE Express and then fixed with 4% paraformaldehyde/PBS for 15 min at room temperature. After washing with PBS, cells were permeabilized with 0.4% Triton X-100/PBS and blocked with 3% BSA for 10 min. About 1–3 × 10^6^ cells were stained for 30 min with directly conjugated antibodies against Krt14 (1:5000, Rabbit, monoclonal, EPR17350), CD200 (5 μL/10^6^, Mouse, monoclonal, ab134489), and ITGA6 (5 μL/10^6^, mouse, monoclonal, ab30497) (Abcam) at room temperature. Control samples were stained with isotype-matched control antibodies (Abcam). After washing with PBS, cells were sequentially resuspended in FACS buffer and then processed for analysis on a Fortessa Flow Cytometer (BD Biosciences, Franklin Lakes, NJ).

Quantitative RT-PCR was used to examine gene expression of iPSC-derived EpSCs, human hair follicle stem cells (HFSCs, 3600708, Celprogen, Shanghai, China) and the iPSCs. Quantitative RT-PCR was performed with SYBR® Green Realtime PCR Master Mix (TOYOBO, Shanghai, China). A 0.5 μL sample of cDNA was used per reaction. All reactions were performed in an ABI 7500 Fast Machine and analyzed by ABI Relative Quantification Study software (Applied Biosystems, Foster City, USA). The RT-PCR results were confirmed by at least three independent analyses. Relative gene expression was calculated using the ∆∆CT method and showed a fold change from iPSCs. The housekeeping gene GAPDH was used as an internal reference. The primer sets used for RT-PCR are listed in Table 1.

### Culture of iPSC-derived EpSCs on a hAAM

A human acellular amniotic membrane (hAAM) was prepared by a previously described method [29]. Briefly, the placenta was collected with ethical approval within 24 h after natural delivery and stored at 4 °C. To prepare the hAAM, the amnion was separated from the chorion by blunt dissection and washed three times with PBS to remove the blood and cellular debris. After incubation in 0.1% EDTA at 37 °C for 2 h, the epithelium was gently denuded with a cell scraper. Subsequently, the acellular amniotic membrane was air dried overnight on a platform, cut into 10 × 10 cm squares, sterilized, and stored at 4 °C.

After rehydration for 24 h in PBS, the hAAM was cut into 2 × 2 cm pieces, placed in 6-well plates, rinsed in a 0.1% gelatin for 30 min, and incubated with a laminin solution (0.1 mg/mL in D-KSFM, Thermo Fisher) for several hours at room temperature. Then, the hAAM was used immediately as a scaffold to culture iPSC-derived EpSCs. Briefly, cells differentiated for 11 days were separated from the feeder layer by small mass and washed three times through sedimentation to remove the contaminating cells from the feeder layer. Then, the differentiated cells were seeded on the hAAM at a density of 1 × 10^5^ cells/cm^2^ and then cultured under the 3T3 condition with D-KSFM medium containing Y-267232. The medium was changed every other day. After 4 days of culture, cell adhesion and growth were examined by H&E staining. Cells cultured in tissue culture plates precoated with laminin were used as controls.

For proliferation analysis, a laminin (60 μg/mL) (Sigma) solution was added to each well of a 96-well plate and incubated at room temperature for several hours. A hAAM was rehydrated with D-KSFM and then transferred to the 96-well plate. iPSC-derived EpSCs were seeded in the plate at a density of 2 × 10^4^ cells per well and incubated at 37 °C with 95% humidity for 24 h. The medium was renewed every other day, and cytocompatibility was tested by a CCK-8 kit (Meilunbio, Dalian, China) at given times.

Scanning electron microscopy (SEM) was used to examine the morphology of cells on the hAAM. A hAAM with iPSC-derived EpSCs was stored at − 80 °C for 1 h and then freeze dried under a vacuum for 24 h. Then, iPSC-derived EpSCs cultured on the hAAM were gently washed with PBS, fixed for 48 h using 2.5% (*w*/*v*) glutaraldehyde (Sigma-Aldrich), postfixed in 0.1% (*w*/*v*) osmium tetroxide (Sigma-Aldrich) overnight at 48 °C, and finally dried. Subsequently, a hAAM alone or hAAM with iPSC-derived EpSCs were mounted on aluminum stubs and coated with gold before examination by a scanning electron microscope (Tecnai G2 F30, FEI, USA).

### Repair of full-thickness skin defects in nude mice with iPSC-derived EpSCs on a hAAM

The animal experiments were approved by the Institutional Animal Care and Use Committee at Zhejiang Chinese Medical University (code: 10296). Six-week-old healthy BALB/C nude mice (Slack, Shanghai, China) weighing 20–30 g were used for the following experiments. After 10% chloral hydrate abdominal anesthesia and iodophor disinfection, two 1.5 × 1.5 cm areas were marked on the back of each side of a nude mouse, and deep wounds were cut along the markers into the fascia.

According to the different methods of wound treatment, 15 BALB/C-nu mice were randomly divided into three groups. In each group, two wounds in a mouse were treated by different methods. Briefly, a hAAM with cells in comparison with a hAAM alone was used in group 1, a hAAM alone in comparison with Vaseline was used in group 2, and a hAAM with cells in comparison with Vaseline was used in group 3. Accordingly, the wounds were respectively treated by covering with Vaseline (blank) or treated by a hAAM (hAAM) or transplanting a hAAM with iPSC-derived EpSCs (hAAM with cells). Specifically, the wounds were gently rinsed with gentamycin in a saline solution, covered with Vaseline, a hAAM, or hAAM with cells, and then the wounds covered with a hAAM or hAAM with cells were dressed with Vaseline. Finally, all wounds were covered with normal saline gauze and bandaged with an elastic bandage. The healing time of wounds was analyzed after transplantation.

Immunohistofluorescence staining was conducted to examine the expression of cytokeratin 15 (Krt15), cytokeratin 10 (Krt10), and loricrin in the healing skin. At day 28 after treatment, nude mice were sacrificed, and the healing skin was harvested to prepare an OCT-embedded specimen. The specimen was cut into frozen sections of 10 μm in thickness. After fixation with cold acetone, the frozen sections were preincubated in a 5% normal goat serum for 10 min and then incubated with a mouse polyclonal anti-Krt15-specific antibody (1:50, ab80522), anti-CK10-specific antibody (1:200, ab76318), and anti-loricrin-specific antibody (1:200, ab85679) (Abcam) at 4 °C for 16 h. Then, the frozen sections were incubated for 40 min at room temperature with Alexa-conjugated fluorochrome 647 (1:200, Abcam), counterstained with DAPI (Sigma-Aldrich), and observed under a fluorescence microscope (LSM710, ZEISS). Additionally, a part of the skin from the same site was fixed in 4% paraformaldehyde, stained with H&E, and observed for skin structures and skin appendages under an optical microscope.

### Statistical analysis

Statistical differences were assessed by a one- or two-way analysis of variance or the two-sided Student’s *t* test. A *P* value of less than 0.05 was considered significant. All statistical analyses were performed using GraphPad PRISM version 7.04.

## Results

### Generation and characterization of iPSCs

**Fig. 1:**
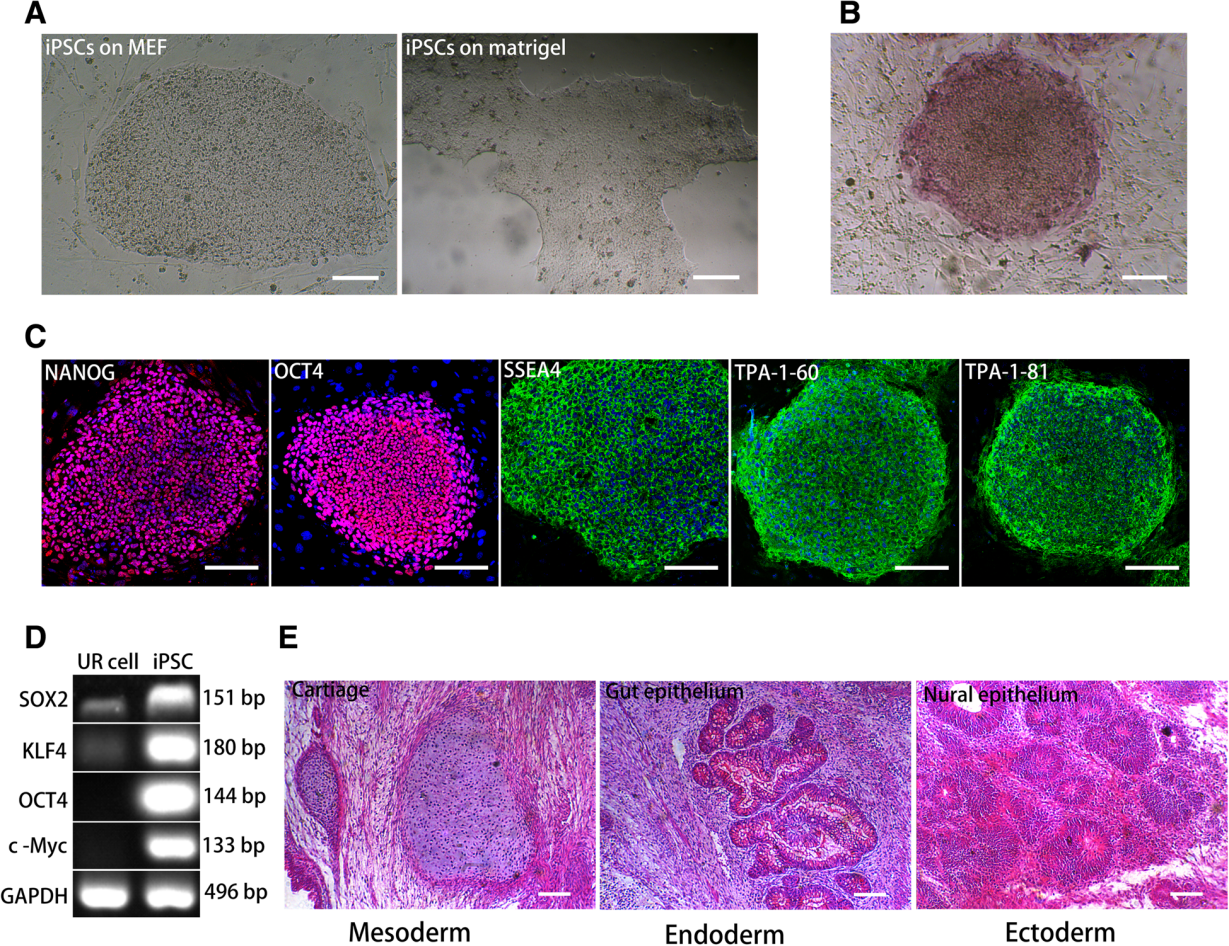
Characterization of induced pluripotent stem cells (iPSCs) derived from urinary cells. **a** iPSCs exhibiting ESC-like morphology in coculture with mouse embryonic feeder fibroblasts or in culture with mTeSR1. Scale bar, 100 μm. **b** Alkaline phosphatase staining of iPSCs. Scale bar, 100 μm. **c** Immunofluorescence staining for expression of OCT4, NANOG, SSEA4, TRA-1-81, TRA-1-60, and SSEA4 in iPSCs. Nuclei were counterstained with 4′, 6-diamidino-2-phenylindole (DAPI; blue). Scale bar, 200 μm. **d** PCR assays for expression of OCT4 (endo), SOX2 (endo), KLF4 (endo), and c-Myc (endo) in iPSCs and parental urinary cells. **e** H&E staining of teratomas from NOD-SCID mice showing gut epithelium in the endoderm, neural epithelium in the ectoderm, and cartilage in the mesoderm. Scale bar, 100 μm

iPSCs were generated from urinary cells using EBNA1-based episomal vectors. At 25 days after transfection, iPSC colonies with the characteristic morphology of human embryonic stem cells were formed as shown in Fig. 1a. Similar to ESCs, iPSC colonies were significantly positive for alkaline phosphatase (Fig. 1b). Pluripotency markers, including nuclear transcription factors POU class 5 homeobox 1 (OCT3/4), NANOG homeobox (NANOG), TRA-1-81, and TRA-1-60, were detected in iPSCs by immunostaining (Fig. 1c), suggesting that iPSCs expressed the same specific marker proteins as embryonic stem cells. Activation of endogenous pluripotency genes SOX2, KLF4, OCT4, and c-MYC was confirmed by PCR (Fig. 1d) compared with urinary cells. The pluripotency of iPSC clones was further validated by a teratoma formation test after injection of iPSCs into NOD/SCID mice. The results showed differentiation of iPSCs into teratomas with the characteristic three germ layers: gut epithelium (endoderm), cartilage (mesoderm), and neural epithelium (ectoderm) (Fig. 1e). These results indicated that pluripotent stem cells with specific gene expression and differentiation pluripotency resembling those of embryonic stem cells had been established.

### Differentiation and characterization of iPSC-derived EpSCs

**Fig. 2:**
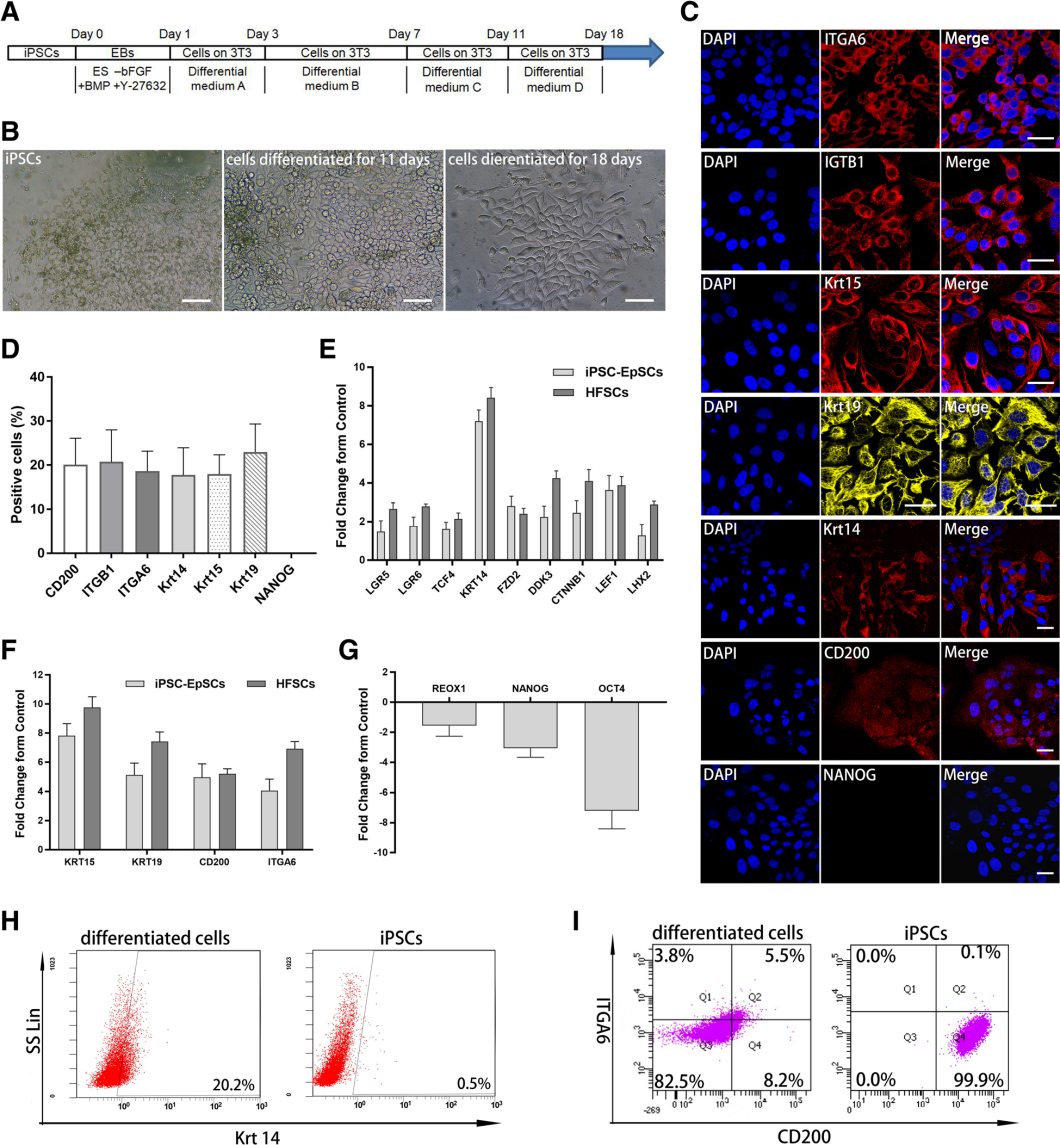
Generation and characterization of iPSC-derived EpSCs. **a** Schematic of the EpSC differentiation showing the presence of retinoic acid, BMP-4, and EGF at different times. **b** Morphology of cells at different stages of differentiation: iPSCs, high-density polygonal morphology of cells differentiated for 11 days, and paving stone morphology of cells differentiated for 18 days. Scale bar, 50 μm. **c** Percentage of positive cells were 20.73 ± 7.26%, 20.05 ± 6.02%, 18.57 ± 4.59%, 17.71 ± 6.19%, 17.90 ± 4.42%, 22.94 ± 6.37%, and 0.01% ± 0.01% for ITGA6, CD200, Krt14, Krt15, ITGB1, Krt19, and NANOG respectively (*n* = 5, mean ± SD). **d** Immunofluorescence analysis showing positive expression of CD200, Krt15, Krt19, ITGB1, and ITGA6 and negative expression of NANOG in differentiated cells in comparison with iPSCs. Scale bar, 30 μm. **e**–**g** RT-PCR analyses of epithelial stem cell-related genes (LGR5, LGR6, TCF4, FZD2, DDK3, CTNNB1, Krt14, LEF1, and LHX2) (**e**), hair follicle stem cell-related genes (CD200, Krt15, Krt19, and ITGA6) (**f**), and pluripotent genes (NANOG, OCT4, and REOX1) (**g**) in cells induced for 11 days compared with control hair follicle stem cells (hHFSCs) and iPSCs. The housekeeping gene GAPDH was used as an internal reference. Error bars represent the S.D. (*n* = 3). **h** Flow cytometric analysis of Krt14 in iPSC-derived EpSCs in comparison with iPSCs. **i** Flow cytometric analysis of CD200 and ITGA6 in iPSC-derived EpSCs in comparison with iPSCs

We induced iPSCs to differentiate into EpSCs according to a published protocol [26] as shown in Fig. 2a. iPSCs were pretreated with BMP-4 for 1 day to block the neural fate and then plated onto mitomycin-C-treated 3T3 fibroblasts in the presence of RA for 2 days to form ectodermal like cells. These cells were induced further for 8 days to differentiate into EpSCs in the presence of RA, BMP-4, and EGF, followed by final expansion of the epithelial lineages for 7 days in the presence of BMP-4 and EGF. At day 11 of differentiation, differentiated cells were observed with a high density of polygonal morphology (Fig. 2b). After culture in differentiation medium D for another 7 days, we acquired iPSC-derived epithelial cells with paving stone morphology (Fig. 2b). However, the number of cells showed a decreasing tendency during culture in differentiation medium D. In addition, it was easier to remove the contaminating cells in feeder layer from cells differentiated for 11 days. Therefore, cells at 11 days of differentiation were collected and used for the following experiments.

Immunofluorescence analysis was performed to show expression of markers specific for epithelial cells (ITGB1) and hair follicle stem cells (CD200, ITGA6, Krt14, Krt15, and Krt19) in these cells (Fig. 2c). The results showed that the positive expression were 20.73% ± 7.26% (ITGB1, *n* = 5), 20.05% ± 6.02% (CD200, *n* = 5), 18.57% ± 4.59% (ITGA6, *n* = 5), 17.71% ± 6.19% (Krt14, *n* = 5), 17.90% ± 4.42% (Krt15, *n* = 5), and 22.94% ± 6.37% (Krt19, *n* = 5) respectively, but the pluripotency gene NANOG was barely detected (0.01% ± 0.01%, *n* = 5) (Fig. 2c, d). RT-PCR analysis showed high expression of genes specific for epithelial stem cells (LGR5, LGR6, TCF4, FZD2, DDK3, CTNNB1, Krt14, LEF1, and LHX2) (Fig. 2e) and hair follicle stem cells (CD200, Krt15, Krt19, and ITGA6) (Fig. 2f), and downregulation of pluripotency genes (REOX1, NANOG, and OCT4) (Fig. 2g). These results were significantly different from iPS cells, but similar to follicle stem cells derived from human hair follicle bugles, indicating that cells differentiated into cells expressing epithelial stem cell markers and hair follicle stem cell markers after 11 days of induction.

Krt14 was a marker of epithelial stem cells, and CD200^+^/ITGA6^+^ cells were defined as folliculogenic epithelial stem cells [26]. At day 11 of differentiation, we collected clumps of cells by dispase II treatment and performed flow cytometry to analyze Krt 14^+^ and CD200^+^/ITGA6^+^ cells. The results showed that 20.2% of cells were Krt14^+^, and 5.5% of cells were CD200^+^/ITGA6^+^ in comparison with the undifferentiated iPSCs (Fig. 2h, i), revealing that epithelial stem cells and CD200^+^/ITGA6^+^ cells emerged after differentiation for 11 days.

### Properties of iPSC-derived EpSCs cultured on a hAAM

**Fig. 3:**
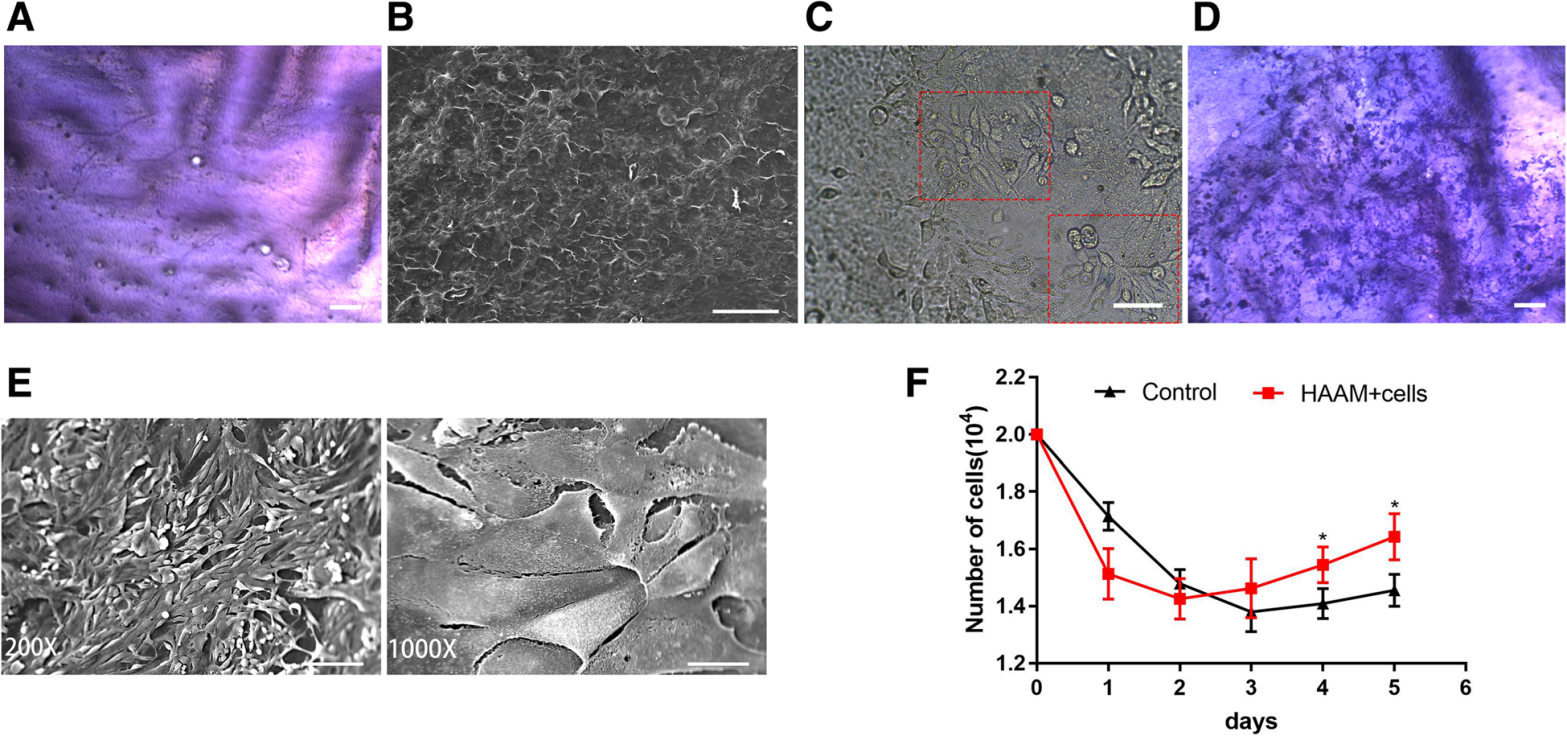
Properties of the hAAM and iPSC-derived EpSCs cultured on a hAAM. **a** H&E staining showing that epithelial cells were removed from the hAAM. Scale bar, 200 μm. **b** Three-dimensional porous morphology of hAAM detected by scanning electron microscopy. Scale bar, 100 μm. **c**, **d** iPSC-derived EpSCs cultured on a hAAM were observed at day 4 after loading by optical microscopy (scale bar, 50 μm) (**c**) and H&E staining (scale bar, 200 μm) (**d**). **e** Attachment of iPSC-derived EpSCs to the acellular amniotic membrane was detected by scanning electron microscopy (SEM). Cells were seeded at 1 × 10^5^ cells/cm^2^. Scale bar represents 100 μm (× 200) and 25 μm (× 1000). **f** CCK8 analysis of iPS-EpSCs cultured on a hAAM showing a higher ability for proliferation in comparison with cells cultured on laminin

A human acellular amniotic membrane (hAAM) separated from the chorion was stained with H&E as shown in Fig. 3a. The hAAM epithelium was transparent, and the epithelium had been removed. The microscopic morphology of the hAAM was examined by scanning electron microscopy (SEM) as shown in Fig. 3b, which showed that the surface of the hAAM had a porous morphology structure. This structure may provide an appropriate environment for cell attachment and proliferation with sufficient nutrients and oxygen [30]. Before seeding onto a hAAM, cells differentiated for 11 days were digested by dispase II and separated into single cells. At day 4 after seeding, cells cultured on the amniotic membrane were observed under an optical microscope (Fig. 3c), which were also stained by H&E (Fig. 3d). Scanning electron microscopy (SEM) showed that cells cultured on the amniotic membrane grew at a high density, and some cells exhibited morphology similar to that of epithelial cells (Fig. 3e).

Subsequently, we examined the proliferation ability of cells cultured on the amniotic membrane using the CCK8 assay. As shown in Fig. 3f, the density of cells cultured on the amniotic membrane decreased gradually during the first 2 days and then increased gradually. The density of cells cultured on laminin decreased before the beginning to increase after 3 days of culture. At day 4 after loading, cells cultured on the amniotic membrane showed a higher proliferative capacity than cells cultured on laminin.

### Repairing effects of a hAAM with iPSC-derived EpSCs on full-thickness skin defects in nude mice

**Fig. 4:**
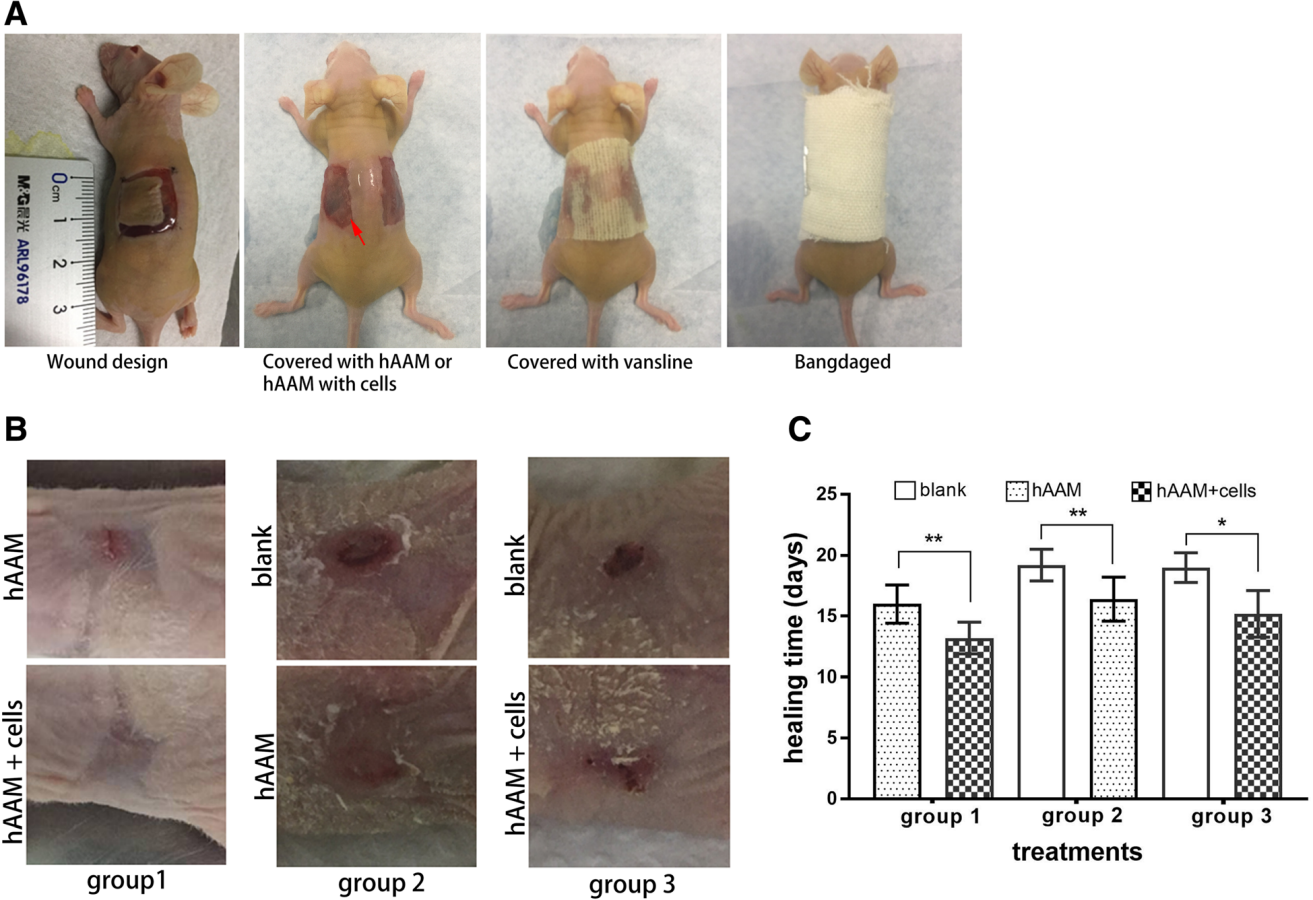
Repair of full-thickness skin defects in nude mice. **a** Procedure of the animal experiment. **b** Healing status of wounds treated for 14 days by various methods. **c** Healing time analysis of wounds in the three groups (*P* ≤ 0.05)

The treatment experiments of full-thickness skin defects in nude mice were performed as shown in Fig. 4a. Mice used for the treatment experiments were divided into three groups, and each mouse had two wounds on each side of its back. In the three groups, two wounds in each mouse were respectively treated by a hAAM with cells or hAAM alone (group 1), a hAAM and Vaseline (group 2), or a hAAM with cells and Vaseline (group 3) (Fig. 4b). The healing time of each wound was analyzed. The results showed that the healing times of wounds were 14 ± 0.7 days (hAAM with cells) and 16.0 ± 1.6 days (hAAM) in group 1, 16.4 ± 1.8 days (hAAM) and 19.2 ± 1.3 days (Vaseline) in group 2, and 15.2 ± 1.9 days (hAAM with cells) and 19.0 ± 1.2 days (blank) in group 3 (Fig. 4c). These results indicated that the hAAM shortened the time of wound healing in comparison with Vaseline, and a hAAM with iPSC-derived EpSCs further accelerated wound healing.

**Fig. 5:**
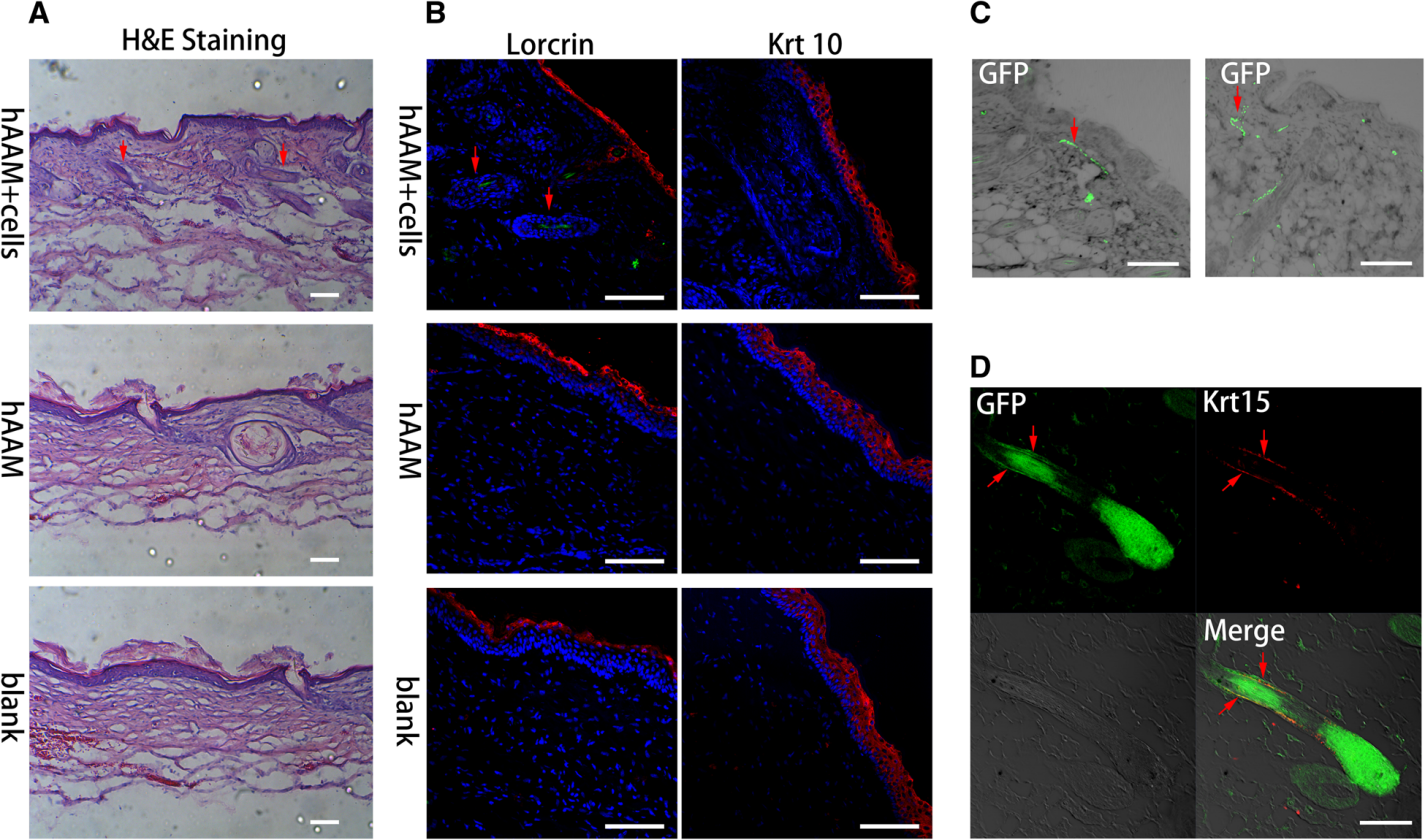
Immunohistochemical and H&E staining analyses. **a** H&E staining of the structures in the healing epidermis. Follicle-like structures were detected in the group of the hAAM with cells in comparison with the group of the hAAM alone and blank group. Scale bar represents 200 μm. **b** Expression of keratinocyte markers Krt10 and loricrin in the upper epidermis. **c** GFP was detected in newly formed hair follicles and epidermis. Scale bar represents 100 μm. **d** Expression of hair follicle-specific marker Krt15 in the outer sheath of hair follicles overlapping with the positive location of GFP. Scale bar represents 100 μm

Nude mice were sacrificed at day 28 after treatment. The healing epidermis was harvested, fixed in 4% polyoxymethylene, and then stained with H&E. The H&E staining showed many typical hair follicle-like structures (Fig. 5a) in the healing epidermis treated with the hAAM with cells compared with those in treated with the hAAM alone and Vaseline (Fig. 5a).

Immunohistochemical analysis revealed the expression of mature keratinocyte marker Krt10 and loricrin in the upper epidermis of healing skin (Fig. 5b). Some cells in the newly formed epidermis were positive for GFP (Fig. 5c), revealing that iPSC-derived EpSCs participated in the reconstruction of the interfollicular epidermis. Expression of the marker Krt15 specific for hair follicles was also detected in newly formed hair follicles (Fig. 5d). The location of Krt15 expression was the “bulge” where the hair follicle stem cells were located. More importantly, the expression of GFP, which was pretransfected into iPS cells, was also detected in the positive location of Krt15 (Fig. 5d), suggesting that cells derived from iPSCs also participated in the formation of follicles with a bulge.

## Discussion

Pluripotent stem cells can differentiate into various kinds of cells under appropriate conditions that involve many growth factors. Bone morphogenetic protein 4 (BMP-4) promotes differentiation of the epidermis and inhibits neural fate through the Smad pathway [31, 32]. Therefore, it is used to induce embryonic stem cells to differentiate into keratinocytes [33]. Moreover, retinoic acid and EGF activate expression of both ectodermal and mesodermal markers, which are used to induce differentiation of ectodermal and mesodermal cells [34]. After establishing the neuroectoderm fate, RA is commonly used to induce nerve differentiation [35]. However, when the neuronal fate is initially blocked by BMP-4, RA induces the epithelial fate of ectodermal derivatives (presumably through induction of ΔNp63 expression) [36], thereby significantly improving the differentiation efficiency of keratinocytes. BMP-4 and RA signaling pathways have been used to induce iPSCs into keratinocytes [23]. EGF was subsequently added [37], which was found to significantly improve the efficiency of keratinocyte amplification [38] and provided sufficient cells for cell-based therapy of skin wounds. Yang et al. [26] further found that they maintained the differentiation of iPS cells at the early stage of epithelial differentiation by precisely regulating the EGF signaling pathway, and then obtained multipotent epidermal stem cells expressing markers ITGA6 and CD200. In the present study, we established an iPSC line derived from urinary cells of a healthy donor through activation of endogenous pluripotent genes SOX2, KLF4, OCT4, and c-MYC and induced iPSCs to differentiate into epithelial stem cells (iPSC-derived EpSCs) expressing CD200 and ITGA6 genes specific for hair follicle stem cells. We cultured small clumps of iPSCs on a low-cluster plate to form EBs and blocked the neural fate of iPSCs with BMP-4. The newly formed EBs were subsequently seeded onto mitomycin-C-treated 3T3 fibroblasts in the presence of RA for differentiation of ectodermal like cells and cultured for 8 days with RA, BMP-4, and EGF to induce these cells to differentiate into epidermal stem cells. After 11 days of differentiation, we obtained cells with epithelial-specific polygonal morphology. These cells expressed both genes specific for epithelial stem cells (Krt14, Krt1, and ITGB1) and hair follicle stem cells (Krt15, Krt19, CD200, and ITGA6). Moreover, some of these cells were CD200+/ITGA6+, which may be defined as folliculogenic epithelial stem cells with properties that resembled hair follicle stem cells in a previous study [26].

The hAAM is the innermost layer of the placental membranes, consisting of an epithelial cell layer, basement membrane, and acellular compact layer [39]. As a readily available natural material with abundant collagen and low immunogenicity [40], the hAAM has been used as a temporary dressing to cover wounds or more commonly used in ophthalmic surgery [41, 42], which has been reported to reduce inflammation, promote epithelialization, and prevent scarring [41, 42]. After denuding of amniotic epithelial cells, the hAAM further eliminates immunological rejection while retaining collagen [30], making it a promising scaffold for cell attachment and proliferation [27]. A variety of cell types has been cultured on the amniotic membrane (keratinocytes, mesenchymal cells, and fibroblasts) to repair cartilage, liver, and trachea [43–46]. In a study by Sanluis-Verdes et al. [47], fibroblasts and epidermal stem cells were cultured on each side of an acellular amniotic membrane to form a skin substitute with both dermal and epidermal structures. Wu et al. [48] reported the use of adipose-derived mesenchymal stem cells cultured on an acellular amniotic membrane to repair skin defects in nude mice. Amniotic membranes have shown great potential as scaffolds for tissue engineering. However, there have been no reports of iPSC-derived cells cultured on an amniotic membrane. The purpose of this study was to prepare a skin substitute consisting of a hAAM and iPSC-derived EpSCs for use as a skin substitute to repair skin defects in nude mice and evaluate its effects on wound repair, as well as illustrating the role of iPSC-derived EpSCs as seed cells in a skin substitute for skin repairing. During the culture of iPSC-derived EpSCs on the hAAM, we found that the number of cells seeded on the hAAM decreased gradually in the first 2 days of culture, but gradually increased thereafter. It has been inferred that a decrease of cells in the first 3 days is due to apoptosis of non-epithelial cells in D-KSFM medium without RA. SEM analysis of cells cultured on a hAAM for 4 days showed the morphology of iPSC-derived EpSCs cultured on a hAAM, which indirectly confirmed the cytocompatibility of the amniotic membrane as a scaffold for a skin substitute. Subsequently, the composite of the hAAM and cells was transplanted onto full-thickness skin defects in nude mice to evaluate the effects of this composite on wound repair. The results showed that the healing time of the wounds treated by the hAAM with cells was significantly shorter than that wounds treated by the hAAM and Vaseline, suggesting that the treatment with the composite of the hAAM and cells accelerates wound healing. We also found that new hair follicle structures were formed in healing wounds treated with the composite of the hAAM and cells, which were rarely detected in wounds repaired by the hAAM and Vaseline. In addition, GFP was detected in the newly formed epidermis and hair follicles of wounds treated with the composite of the hAAM and cells. These results suggest that the skin substitute consisting of the hAAM and iPSC-derived EpSCs promotes wound healing and participates in the formation of skin appendages.

However, we found that apoptosis of the induced cells was increased gradually during the subsequent 8 days of culture after the induction of iPSCs for 11 days of differentiation, although many cobblestone-like clones were observed. This is consistent with phenomena observed in the previous study [25] that showed a restricted proliferative capacity of ESC-derived keratinocytes, which was different from that of postnatal keratinocytes. Therefore, further studies are needed to improve the proliferation stability and efficiency of differentiated cells. In addition, how to keep amniotic membranes moist is still a challenge to repair wounds. Despite the use of saline gauze in our experiments, the amniotic membranes formed a hard membrane covering the wound at day 7 after implantation. Although there was a significant difference in the healing time of the hAAM with cells from that of the hAAM alone, the formation of a hard membrane may reduce wound healing. Although GFP was detected in the bulge, it was uncertain that the induced iPSCs did generate the hair follicle stem cells in vivo. Therefore, GFP^+^ cells purified from the healed wound should be performed another round of skin transplantation to distinguish the HFSCs to transient-amplifying cells.

## Conclusions

In conclusion, we obtained CD200^+^/ITGA6^+^ epithelial cells by inducing urine cell-derived iPSCs through regulating retinoic acid, BMP-4, and EGF signaling pathways. After the combination of the hAAM with iPSC-derived EpSCs to form a skin substitute, the role of EpSCs in the reconstruction of hair follicles was confirmed. Therefore, iPSC-derived EpSCs used as seed cells to construct skin substitutes are potential candidates to repair skin defects, reconstruct skin appendages, and recover skin functions.

## Abbreviations

BMP: Bone morphogenetic protein
DKSF: Defined keratinocyte serum free
DMEM: Dulbecco’s modified Eagle’s medium
EBs: Embryoid bodies
EGF: Epidermal growth factor
EpSCs: Epithelial stem cells
ESC: Embryonic stem cell
F12: Ham’s F-12 Nutrient Mixture
GFP: Green fluorescent protein
hAAM: Human acellular amniotic membrane
iPSCs: Induced pluripotent stem cells
ITGA6: Integrin α6
ITGB1: Integrin β1
Krt: Cytokeratin
MEF: Mouse embryonic fibroblast
PBS: Phosphate-buffered saline
PCR: Polymerase chain reaction
RA: Retinoic acid
REBM: Renal epithelial basal medium
REGM: Renal epithelial cell growth medium
RT-PCR: Reverse transcription polymerase chain reaction
SEM: Scanning electron microscopy
